# A comparison of the relationship between parental efficacy and social support systems of single teen mothers across different family forms in South African low socioeconomic communities

**DOI:** 10.1186/s12905-021-01300-w

**Published:** 2021-04-17

**Authors:** Samantha L. Coert, Babatope O. Adebiyi, Edna Rich, Nicolette V. Roman

**Affiliations:** Centre for Interdisciplinary Studies of Children, Families and Society, Faculty of Community and Health Sciences, University of the Western Cape, Robert Sobukwe Road, Bellville, Cape Town, 7535 South Africa

**Keywords:** Teen parenting, Teen mothers, Parental efficacy, Family form, Social support, Teenage pregnancy

## Abstract

**Background:**

Teenage parenting is recognised as one of the greatest health and social problems in South Africa. Research in South Africa has shown that by the age of 18 years, more than 30% of teens have given birth at least once. Teen mothers may feel disempowered because they are ‘othered’ and consequently, may develop forms of resistance which in most cases may inhibit their ability to parent. Social support is therefore, an imperative intervention for successful teen parenting but this is not clearly understood in South Africa. This study aimed to compare the relationship between parental efficacy and social support systems of single teen mothers across different family forms.

**Methods:**

A quantitative methodology with a cross-sectional comparative correlation design was conducted with 160 single teen mothers who resided with a family in a low socio-economic community. The participants completed a self-report questionnaire that comprised of the Social Provisions Scale, and the Parenting Sense of Competence scale. Descriptive statistics and Pearson correlation were used to investigate the data.

**Results:**

A significant positive relationship between social support and parental efficacy was found. When comparing different family forms, single teen mothers’ residing with one parent reported greater levels of parental efficacy and single teen mothers’ residing with two parents, re-counted high levels of social support under the subscales; guide, reliable and nurture. However, when computing for guardian-skip generation, results show that there is no significant relationship between parental efficacy and social support. As well as no correlation across subscales of social support.

**Conclusion:**

The positive relationships between social support and parental efficacy are important for planning and applying parenting programmes amongst single teen mothers and facilitating awareness regarding the importance of social support and family forms when considering parenting practices.

**Supplementary Information:**

The online version contains supplementary material available at 10.1186/s12905-021-01300-w.

## Background

Parental efficacy acts as one of the most powerful predictors of future success, as it not only plays a part in the goals a person sets in parenting and which activities that person becomes involved in, but also influences the coping strategies the person will adopt under difficult circumstances [1]. According to Coleman and Karraker [2], parental efficacy refers to the parent’s expectations of competence in the role as parent.

On the other hand, teenage pregnancy is a universal phenomenon affecting both developed and developing countries [3, 4], with approximately 16 million births to mothers aged 15–19 years, and two million to girls under the age of 15 years, annually [5]. In South Africa, 39% of 15- to 19-year old girls have been pregnant at least once [6], the majority, unplanned [7, 8] and increasing amongst historically classified Coloured females [9]. One thousand one hundred and sixty-two women under the age of 19 were pregnant in different schools in Elsies River, Macassar, Bishop Lavis, Hanover Park and Vanguard around the Western Cape during just seven months in the year 2004 [10]. Furthermore, Honikman, et al. [11], report on Perinatal Mental Health Project indicated that 49% of teen mothers are pregnant again within 24 months. During the literature review it becomes clear that there is a lack of research examining the kind of support received by teen mothers, more so historically classified Coloureds [9].

Findings of a report focusing on the needs of teenage parents suggested that these teen girls face various challenges related to stigma from peers, community members as well as their family, while the men who have impregnated these girls often deny responsibility [12]. Without the required support and restricted opportunities to complete their education; these teen mothers find themselves susceptible to a number of risks for example malnutrition, poverty and the possibility of developing poor parental efficacy which would all impact on her development as a teen mother as well as that of her child. In addition, Rafferty, Griffin and Lodise [13] and Mollborn and Dennis [14] found that unmarried teenage mothers are more vulnerable than married ones because in many cases the unmarried pregnant girls are rejected by their parents as they have added shame and an additional burden on the family.

Amoateng et al. [15] argue that there is a general consensus in defining families and the forms in which they present in as, social groups that are related by blood (kinship), marriage, adoption, or affiliation with close emotional attachment to each other that endure over time and go beyond a particular physical residence. A synopsis of South African family forms includes; nuclear families, extended families, child-headed households, single parent families and multi-generational families [16].

The interaction between parental efficacy beliefs, parenting and social support is likely to vary by environmental and family contexts [17]. According to Amoateng et al. [15] families operate as a central form of social support to individuals, in addition to forming an intrinsic component of collective networks and ecologies. With a decreased support system, single teen mothers may find themselves lowering their evaluation of the quality of their parenting; as exposure to what quality parenting entails might be low. This is because the size of a social support system plays a role in the quality of parenting [18].

According Van Den Berg [18], greater satisfaction with support networks may result in a greater sense of parental efficacy for teen mothers. Also, Michalos [19] asserts that social support from family members plays a role in the facilitation of teen mother’s parental efficacy across the sphere of her parenting role. However, there is paucity of research on the relationship between social support and parental efficacy, particularly in low socioeconomic communities. The current study aims to make a contribution to existing knowledge on teenage parenting and family functioning by determining and comparing the relationship between parental efficacy and social support of single teen mothers in different family forms.

## Methods

### Study design

The current study used a quantitative methodological approach with a cross-sectional comparative study design. For this study, the correlation design was necessary to determine if a relationship between parental efficacy and social support systems exist. Whereby, the comparative design was applicable to examining the differences in relation to parental efficacy and the support received from the different family forms of single teen mothers.

### The study community and sample

The study was conducted in four low socioeconomic communities across the Western Cape (Mitchells Plain, Elsies River, Factreton and Bishop Lavis) [20, 21]. These areas were selected because of the high concentration of teenage pregnancy, low levels of skills and education, high levels of unemployment, poverty and substance abuse within them. The initial sample size was 320 single teen mothers based on the suggestion by Suhr [22] and Hatcher [23]. Eligibility criteria was set as:—(1) be a single teen mother, (2) have given birth between January 2009–January 2015, (3) single mothers who were aged between 13 and 19 years when they had their first child, (4) single teen mothers should for a period of one year have resided with or is currently residing with family, caregiver or members thereof and (5) the child should be age 5 and younger. The study made use of a convenience sampling procedure, so as to ensure that subgroups within the broader community population would be adequately represented in the sample.

## Research instrument

This study used two instruments to measure the variables under study. The first instrument was the Social Provisions Scale (SPS) as developed by Cutrona and Russell [24] in 1987. The SPS is a 24-item measure that provides six subscales, Reliable Alliance; Attachment; Guidance; Nurturance; Social Integration; and Reassurance of Worth. The original version of the scale uses a Likert response format. The SPS examines the degree to which participant’s social relationships provide various dimensions of social support.

Total internal consistency reliability for the Social Provisions scales is excellent (α = 0.93) with alpha coefficients for the total sample ranging from 0.59 (Opportunity for Nurturance) to 0.78 (Guidance) on the individual scales. Total scale alpha reliabilities are excellent when considered by caregiver race (α = 0.91–0.95) and study site (α = 0.90–0.93). The scale has been used within African American samples, including low-income mothers [25, 26].

The second instrument was the Parenting Sense of Competence Scale (PSOC) as developed by Gibaud-Wallston and Wandersman [27] in 1978. The PSOC is a 17-item scale, with 2 subscales. Each item is rated on a 6-point Likert scale anchored by 1 = “Strongly Disagree” and 6 = “Strongly Agree”. Nine (9) items (2, 3, 4, 5, 8, 9, 12, 14 and 16) on the PSOC are reverse coded. The PSOC is used to measure parents’ satisfaction with parenting and their self-efficacy in the parenting role. Factor analysis had revealed two sub-scales within the entire measure: the skill-knowledge scale and value-comforting scale [28]. Gibaud-Wallston and Wandersman [27] examined this measure with parents of infants and found good internal consistency for both scales (0.70 and 0.82 respectively). The alpha for the full scale was 0.79. The two factors remained and were renamed “Efficacy” and “Satisfaction” [29]. Efficacy, the degree to which the parent feels capable, had an alpha of 0.76 and Satisfaction, an affective measure targeting feelings of frustration, anxiety and motivation, had an alpha of 0.75 [28].

### Data collection procedure

The researcher approached a social auxiliary student to assume the role of fieldworker for the study. The fieldworker was employed and trained accordingly to the questionnaire and consenting procedure. The fieldworker made initial contact with possible participants as she had good knowledge of the communities as well as established relationships with teenagers as a result of her previous work undertakings within these communities. After the fieldworker had secured a participant, contact would be initiated with the researcher. The researcher met with the participants to explain the process and objectives of the study. This was followed by the process of participant consent and completion of the questionnaire administered by the fieldworker.

### Data analysis

Data was coded, cleaned and checked for errors. Analyses were conducted using IBM® SPSS® Statistics Version 23.0.0. Descriptive statistical analyses were conducted. Measures of central tendency and measures of dispersion were used to look at the data of each scale and subscale. Since the emphasis of this study is the relationship between quantitative variables, Pearson’s correlation coefficient was used to explore the extent of linear relationships among the variables, and to quantify the strength and direction of the relationship. Cronbach’s alpha coefficients were computed to determine the internal consistency for each measure and relevant subscales. Pearson’s coefficient was used to explore the relationship between the independent variable (family forms & social support) and the dependant variable (parental efficacy).

### Ethics statement

This study received the necessary approval from the research ethics committee of the University of the Western Cape (13/9/16). Participants and parents approached and were informed that participation in the study was not mandatory, and that the participant was free to withdraw from participating should they found it necessary. The information sheet, detailing the aim of the study and participants’ roles, was explained to them. The participants were asked to sign a consent form if they agreed to participate. All the ethical principles were adhered to during the data collection process.

## Results

This section provides the results of the statistical analysis conducted for the study. The results are presented as (1) descriptive information about single teen mothers, and parental efficacy (2) the relationship between the variables, and (3) the comparison of the variables between the different family forms (groups). The Statistical Package for the Social Sciences 23 (SPSS) was used in all the statistical calculations.

### Challenges faced during participants’ recruitment

The study sample was initially set for 320 single teen mothers. Due to the challenges of recruiting this number had to be adjusted to 160. Firstly, the stigma attached with teenage pregnancy is very overwhelming and a problem within our Coloured communities, this created a constant barrier in trying to source possible participants. Secondly, when requiring parental consent from prospective participants’ parents, either the participant herself was not comfortable with involving her parent/parents in the study or the parent refused to give consent, as they wanted nothing to do with the concept of their daughters being a teen mother. Thirdly, one of the Non-Government Organizations (NGOs) contacted to assist with the recruitment of participants closed down due to unforeseen circumstances.

### A description of single teen mothers

Table 1 provides an overview of the demographic variables of single teen mothers in this study (N = 160) and the childcare situation within the home. Table 2 illustrates participants’ (N = 160) age at the time of the survey, age at birth of first child, number of children in household and the family forms identified by single teen mothers.

**Table 1:** Demographic information of participants and childcare situation within the home

	Variables	N = 160	%
Marital status	Married	4	2.5
	Living together/not married	19	11.9
	Single, do not live together and is not married	137	85.6
Race	Coloured	158	98.8
	Black/African	2	1.3
Home language	Afrikaans	145	90.6
	English	13	8.1
	IsiXhosa	2	1.3
Living arrangements	One parent	65	40.6
	Two parent	51	31.9
	Extended (includes partner & partner’s family)	25	15.6
	Guardian-Skip generation	11	6.9
	Alone	8	5.0
Educational level	Primary Schooling	26	16.3
	Secondary Schooling	113	70.6
	Tertiary Schooling	1	.6
	Completed Grade 12 (Matric)	20	12.5
Employment status	Employed	38	23.8
	Unemployed	122	76.3
Variable	N = 160	%	
Childcare situation in your home	I take care of the child/children full time	119	74.4
	I do not take care of the child/children full time	41	25.6
If NO, the children are in care (day care):	Fewer than 20 h per week	4	2.5
	20 h per week or more	37	23.1
Cared for by another adult in our home	Yes	112	70
	No	48	30
If YES, who cares for them	Aunt	7	4.3
	Sister	17	10.6
	Family friend	13	8.1
	Father of the child	4	2.5
	Foster mother	2	1.3
	Child’s grandmother	64	40
	Nanny	3	1.9
	Neighbour	2	1.3

**Table 2:** An overview of the participants’ (N = 160) age at the time of the survey, age at birth of the first child, number of children in household and the family forms identified by single teen mothers

Characteristics SD	N	%^a^	M
Age at time of survey			
15–20	103	64	19.8
21–26	54	33.7	
27–32	2	1.3	
33–38	1	0.6	
Age at birth of first child			
13–16	83	51.9	16.4
17–19	77	48.1	
Number of children in the household			
1	30	18.8	2.75
2	47	29.4	
3	40	25.0	
4	27	16.9	
5	10	6.3	
6	4	2.5	
7	2	1.3	
Family			
One parent	60	37.5	
Two parents	60	37.5	
Extended	37	23.1	
Blended	1	0.6	
Other	2	1.3	

^a^Percentages do not always equal 100 due to rounding

The results in Table 1 shows that majority of the participants were unmarried [137 (85.6%)]. Of the 160 participants, 158 (98.8%) identified themselves as Coloured. Afrikaans was the dominant home language spoken [145 (90.6%)]. The majority of the participants indicated their living arrangements as staying with one parent [65 (40.6%)]. The highest level of education shown was Secondary Schooling [113 (70.6%)], with the majority of participants being unemployed [122 (76.3%)].

In addition, results in Table 1 shows that 119 participants, (74.38%) single teen mothers take care of their child/children on a full-time basis. The remaining 41 participants (25.6%), child/children are in care (day care). Furthermore, alternative care was also provided by other adult (s) within the home [N = 112 (70%)]. Single teen mothers’ own mothers [64 (40%)] sought to care for the child/children, when she is unable to, a sister [17 (10.6%)], a family friend [13 (8.1%)], an aunt [7 (4.3%), the father of the child [4 (2.5%)], a nanny [3 (1.9%)], a foster mother [2 (1.3%)] and or a neighbour [2 (1.3%)].

The majority of mothers [(N = 52) 32.5%] were aged 17 years, when they had their first child. The youngest participant [(N = 1) 0.6%] to have given birth was age 13 and the oldest was 19 years old [(N = 2) 1.3%]. Most participants [(N = 47) 29.4%] reported on average that 2 children reside within the household. Participants described the family form of their families to come from both a one parent and two parent family, both representing N = 60 (37.5%) respectively. The remaining 25% of the participants saw their family form as extended [N = 37 (23.1%)], blended [N = 1 (0.6%)] or other [N = 2 (1.3%)].

### Descriptive statistics of the variables

Mean (M) and Standard Deviation (SD) for the Social Support (SS) of single teen mothers are presented in Additional file 1: Table S1. Additional file 1: Table S2 displays the mean (M) and standard deviation (SD) for the subscales. The subscales are: attachment (attach), social integration (socintegr), reassurance of worth (reassworth), reliable alliance (reliable), guidance (guide) and opportunity for nurturance (nurture). Additional file 1: Table S3 will present the mean (M) and standard deviation (SD) for the Parental Efficacy (PE) of single teen mothers.

Additional file 1: Table S1 represents the mean and standard deviation for each of the 24 SS items for the perceived Social Support for the total sample (N = 160). A high score indicates a greater degree of perceived support.

Results in Additional file 1: Table S1 indicate that the majority of the participants (N = 160)’ agreed’ (M = 2.81, SD = 0.99) that “there is a trust worthy person they could turn to for advice if they were having problems”. Participants (N = 160) similarly indicated that they agree (M = 2.96, SD = 0.84) “…to have a strong emotional bond with at least one other person”, and “… participants (N = 160) further agreed (M = 2.74, SD = 1.03) …there is someone, I could talk to about important decisions in my life”. Majority participants agreed that (M = 2.73, SD = 0.85) … feel responsible for the well-being of another person. However, most participants appeared to disagree (M = 2.32, SD = 0.87) when asked…*my competence and skills are recognized*.

Additional file 1: Table S2 represents the mean and standard deviation for the Social Provision Subscale: Attachment (Items 2R, 11, 17, and 21R), Social Integration (Items 5, 8, 14R, and 22R), Reassurance of Worth (Items 6R, 9R, 13, and 20), Reliable Alliance (Items 1, 10R, 18R, and 23), Guidance (Items 3R, 12, 16, and 19R) and Opportunity for Nurturance (4, 7, 15R, and 24R) for the total sample (N = 160).

Additional file 1: Table S2 results suggest that the most perceived support across the total sample (N = 160) as Attachment (M = 2.61, SD = 0.64) as reported by single teen mothers. Conversely, single teen mothers indicated Reliable Alliance (M = 2.53, SD = 0.81) to be least supported.

This section of the study provides descriptive statistics which addresses one of the objectives which is to determine the prevalence of parental efficacy of the total sample. Means (M) and standard Deviations (SD) for PE of the total sample (N = 160) parental efficacy, are presented in Additional file 1: Table S3 in order to evaluate this objective.

Additional file 1: Table S3 represents the means and standard deviations of 15 items for the Parental Efficacy for the total sample (N = 160).

The Mean score results in Additional file 1: Table S3 suggest that majority of the participants (M = 2.86, SD = 1.69) perceived themselves as confident when… taking care of a child, are easy to solve once you know how your actions affect your child, an understanding I have acquired. In addition, participants somewhat disagreed (M = 2.35, SD = 1.59) to*…parent is manageable, and my problems are easily solved*. Yet, the scores suggest that the majority (M = 4.09, SD = 1.14) *…find the answer to what is troubling my child, I am the one*.

### Comparisons of groups

*T-*tests were conducted to determine if there were significant perceived differences between (1) parental efficacy and (2) social support received from the different family forms of single teen mothers.

Table 3 represents a comparison of the means scores for each scale and subscale for PE, SS and SS subscales (attachment, social integration, reassurance of worth, reliable alliance, guidance and opportunity for nurturance) across different family form.

**Table 3:** Differences of mean scores for parent efficacy, social support (SS) and SS subscales within the family form: one parent (N = 65), two parents (N = 51), extended (N = 25) and guardian-skip generation (N = 11)

	Mean	Standard deviation	Standard error	95% Confidence interval of the difference		Min	Max
				Lower	Upper		
One parent							
Parental efficacy	3.04	0.76	0.09	2.85	3.23	1.94	5.71
Social support	2.56	0.67	0.84	2.39	2.73	1.17	3.88
Guidance	2.53	0.81	0.10	2.33	2.74	1.00	4.00
Reassurance of worth	2.31	0.73	0.91	2.12	2.49	1.00	4.00
Social integration	2.46	0.76	0.94	2.27	2.65	1.00	4.00
Attachment	2.52	0.65	0.81	2.36	2.69	1.00	4.00
Opportunity for nurturance	2.45	0.78	0.97	2.25	2.64	1.00	4.00
Reliable alliance	2.39	0.83	0.10	2.18	2.59	1.00	4.00
Two parent							
Parental efficacy	3.07	0.57	0.08	2.91	3.23	2.24	4.53
Social support	2.60	0.63	0.89	2.42	2.78	1.33	4.00
Guidance	2.45	0.78	0.10	2.23	2.67	1.00	4.00
Reassurance of worth	2.32	0.66	0.93	2.13	2.51	1.00	4.00
Social integration	2.50	0.68	0.95	2.13	2.70	1.00	3.75
Attachment	2.62	0.63	0.88	2.44	2.80	1.25	4.00
Opportunity for nurturance	2.58	0.80	0.11	2.36	2.81	1.00	4.00
Reliable alliance	2.49	0.78	0.10	2.27	2.71	1.00	4.00
Extended							
Parental efficacy	3.21	0.66	0.13	2.94	3.49	2.00	5.24
Social support	2.90	0.50	0.10	2.69	3.11	1.71	3.96
Guidance	2.91	0.67	0.13	2.62	3.19	1.00	4.00
Reassurance of worth	2.67	0.57	0.11	2.43	2.90	1.25	3.75
Social integration	2.79	0.57	0.11	2.55	3.02	1.50	4.00
Attachment	2.77	0.57	0.11	2.53	3.00	1.25	4.00
Opportunity for nurturance	2.82	0.55	0.11	2.59	3.04	1.50	4.00
Reliable alliance	2.86	0.69	0.13	2.57	3.14	1.25	4.00
Guardian-skip generation							
Parental efficacy	3.81	0.52	0.15	2.45	3.16	1.94	3.35
Social support	2.89	0.50	0.15	2.55	3.23	1.75	3.50
Guidance	2.93	0.71	0.21	2.45	3.41	1.25	3.75
Reassurance of worth	2.36	0.47	0.14	2.04	2.68	1.75	3.25
Social integration	2.79	0.63	0.19	2.37	3.21	1.50	3.50
Attachment	2.81	0.48	0.14	2.49	3.14	1.75	3.50
Opportunity for nurturance	2.86	0.59	0.17	2.46	3.26	1.50	3.25
Reliable alliance	2.95	0.73	0.22	2.46	3.44	1.75	4.00

Table 3 illustrates that single teen mothers residing with one parent (*M* = 3.04, *SE* = 0.09), gained greater levels of parental efficacy. On the subscales of SS, guide (*M* = 2.53, *SE* = 0.10), and reliable (*M* = 2.39, *SE* = 0.10), reported greater levels in social support for single teen mothers residing with one parent.

Table 3 suggests that for single teen mothers residing with two parents, (*M* = 3.07, *SE* = 0.08), greater levels of parental efficacy was experienced. On the subscales of SS, guide (*M* = 2.45, *SE* = 0.10), reliable (*M* = 2.49, *SE* = 0.10), and nurture (*M* = 2.58, *SE* = 0.11) re-counted high levels in social support for single teen mothers residing with two parents.

Table 3 was perceived as single teen mothers residing with extended family, (*M* = 2.90, *SE* = 0.10), SS informed greater levels of support. In addition, the subscales of SS, reassworth (*M* = 2. 67, *SE* = 0.1), socintegr (*M* = 2.79, *SE* = 0.11), attach (*M* = 2.77, *SE* = 0.11) and nurture (*M* = 2.82, *SE* = 0.11) displayed greater levels in social support for single teen mothers residing with extended family.

Table 3 suggest that for single teen mothers residing with guardian-skip generation families, are engaged more with reassurance of worth (*M* = 2.36, *SE* = 0.14) and attachment (*M* = 2.81, *SE* = 0.14) under the subscales of SS.

### ANOVA analysis

Below in Table 4 the output of the ANOVA analysis and whether a statistically significant difference between groups means are presented.

**Table 4:** The output of the ANOVA analysis and whether a statistically significant difference between groups

	Sum of squares	df	Mean square	F	Sig
Total score for parental efficacy					
Between groups	1.523	4	.381	.790	.534
Within groups	74.252	154	.482		
Total	75.775	158			
Total score for social support					
Between groups	3.052	4	.763	1.848	.122
Within groups	63.972	155	.413		
Total	67.023	159			
Guide					
Between groups	5.120	4	1.280	2.087	.085
Within groups	95.082	155	.613		
Total	100.203	159			
Reassworth					
Between groups	2.572	4	.643	1.367	.248
Within groups	72.926	155	.470		
Total	75.498	159			
Socintegr					
Between groups	2.895	4	.724	1.391	.240
Within groups	80.667	155	.520		
Total	83.562	159			
Attach					
Between groups	1.587	4	.397	.942	.441
Within groups	65.282	155	.421		
Total	66.869	159			
Nurture					
Between groups	3.699	4	.925	1.611	.174
Within groups	88.950	155	.574		
Total	92.648	159			
Reliable					
Between groups	6.494	4	1.624	2.572	.040
Within groups	97.849	155	.631		
Total	104.344	159			

df, degree of freedom; F, variation within the samples; Sig, significant

One-way ANOVA for parental efficacy (*F* (4, 154) = 0.790, *p* = 0.534) and social support (*F* (4, 155) = 1.848, *p* = 0.122). The following ANOVA’s represent the subscales of Social Support; guide (*F* (4, 155) = 2.087, *p* = 0.085), reassworth (*F* (4, 155) = 1.367, *p* = 0.248), socintegr (*F* (4, 155) = 1.391, *p* = 0.240), attach (*F* (4, 155) = 0.942, *p* = 0.441) and nurture (*F* (4, 155) = 1.611, *p* = 0.174). The *p* values reported are greater than α level 0.05, thus no statistically significant difference exists. However, ANOVA for subscale reliable (*F* (4, 155) = 2.572, *p* = 0.040), this value is less than 0.05, concluding that a statistically significant difference does exist.

### Determining associational aspects of the variables of the study

This section reports on the correlation scores for PE, SS and SS subscales; GUIDE, REASSWORTH, SOCINTEGR, ATTACH, NURTURE and RELIABLE. A Pearson product-moment correlation was computed to assess these differences.

The results in Table 5 provides an indication that there is a relationship between parental efficacy and social support (*r* = 0.636^**^) within one parent (N = 64), this correlation coefficient is highly significant from zero (P < 0.001). When looking at the variable a bit further, there was also a positive correlation between parental efficacy across all subscales of social support; guide (*r* = 0.596^**^), reassworth (*r* = 0.577^**^), socintegr (*r* = 0.610^**^), attach (*r* = 0.596^**^), nurture (r = 0.597^**^) and reliable (*r* = 0.485^**^) within one parent (N = 64).

**Table 5:** Correlation scores for PE and SS between different family forms

		Total score for parental efficacy	Total score for social support	Guide	Reassworth	Socintegr	Attach	Nurture	Reliable
*One parent*									
Total score for parental	Pearson correlation	1	.636**	.596**	.577**	.610**	.596**	.597**	.485**
	Sig. (2-tailed)		.000	.000	.000	.000	.000	.000	.000
Efficacy	N	64	64	64	64	64	64	64	64
*Two parent*									
Total score for parental	Pearson correlation	1	.598**	.504**	.571**	.546**	.508**	.576**	.582**
	Sig. (2-tailed)		.000	.000	.000	.000	.000	.000	.000
Efficacy	N	51	51	51	51	51	51	51	51
*Extended family*									
Total score for parental	Pearson correlation	1	.730**	.539**	.756**	.679**	.651**	.666**	.550**
	Sig. (2-tailed)		.000	.005	.000	.000	.000	.000	.004
Efficacy	N	25	25	25	25	25	25	25	25
*Guardian-skip generation*									
Total score for parental	Pearson correlation	1	.392	.326	.461	.184	.496	.310	.435
	Sig. (2-tailed)		.233	.328	.154	.589	.121	.354	.181
Efficacy	N	11	11	11	11	11	11	11	11

^**^Correlation is significant at the 0.01 level (2-tailed)

When computing for two parents (N = 51), a positive correlation was indicated for parental efficacy and social support (*r* = 0.598^**^). Furthermore, the results also show that there is a positive relationship across all subscales of social support; guide (*r* = 0.504^**^), reassworth (*r* = 0.571^**^), socintegr (*r* = 0.546^**^), attach (*r* = 0.508^**^), nurture (*r* = 0.576^**^) and reliable (*r* = 0.582^**^) within two parents (N = 51).

The results for extended family (N = 25) indicate a correlation between parental efficacy and social support (*r* = 0.730^**^), this correlation coefficient is highly significant from zero (P < 0.001). Additionally, the results also show that there is a positive relationship across all subscales of social support; guide (*r* = 0.539^**^), reassworth (*r* = 0.756^**^), socintegr (*r* = 0.679^**^), attach (*r* = 0.651^**^), nurture (*r* = 0.666^**^) and reliable (*r* = 0.550^**^) within extended family (N = 25).

When computing for guardian-skip generation (N = 11), results show that there is no relationship between parental efficacy and social support. Furthermore, the results also show no correlation across subscales of social support.

## Discussion

The purpose of this study was to investigate and ascertain whether a relationship between parental efficacy and social support of single teen mothers exist. Furthermore, to determine whether a difference in the relationship is present between the different family forms of single teen mothers.

### Parental efficacy

This study is the first in the Western Cape which has taken parental efficacy as an item of investigation with a sample of single teen mothers into specific consideration. The results of the current study suggest that single teen mother’s own characteristics which is found at the microsystem was her optimistic behaviours, pattern of activities, social roles and the interpersonal relations experienced by the single teen mother, contributed to a high level of parental efficacy, which in turn saw single teen mothers reporting satisfaction in their parenting role.

Teen mothers reported being criticised about their parenting skills and receiving unwanted advice on how to raise their children [30]. However, findings from this study and a study by Reiner Hess et al. [31] indicated that the participants had the necessary skills to be a good mother. Similarly, when it came to troubling situations with their child/ children they were able to find solutions on their own.

Resilient behaviours enabled single teen mothers to see themselves as confident, nurturing and possibly satisfied with their parenting abilities. Previous work found that parents’ perception of competence is important, because it may influence not only parenting but also family dynamics and parental health [32].

### Social support

The results of the current study were similar to that conducted by Baumeister and Leary [33], who’s participants indicated that the presence of stable bonds is responsible for an abundance of positive affect (e.g., feeling good). Furthermore, this study suggested that many of the single teen mothers had at least one trustworthy person within their family that they can turn to for advice when faced with a problem. In addition, this connection was further established when all of the participants agreed in their responses to having a strong emotional bond with at least one person within the family.

This association was noted in Baumeister and Leary [33] study, which suggested that being accepted and included leads to a variety of positive emotions and is related to enhanced psychological well-being through its effects on positive affect and self-esteem. In particular, African American teen mothers reported that support from their mother is the most important source of support during their transition to parenthood [31]. For that reason, support from the family of origin is particularly important in the context of teen parenting [31]. It is without question, that the highest reported perceived social support fell within the subscales; Reliable Alliance and Attachment. For example, this similarity of findings can be seen in a study by Watson [34], who reported that individuals tend to interpret others with whom they have a relationship as more favourable.

### The association between parental efficacy and social support

The correlation between parental efficacy and social support indicated a statistically significant relationship. Further correlations reported positive relationships across all subscales of social support. Therefore, a relationship between parental efficacy and social support of single teen mothers does exist. Also, similar to this result, a study by Young [1] featuring Caucasian and Hispanic mothers proved a correlation between social support and parental efficacy. In addition, a study by Hoven [35] investigating 77 parents of children 2 to 5 years who had not yet started kindergarten, reported a significant positive relationship between social support and parental efficacy. Therefore, an argument can be made that having social support leads to greater satisfaction in the parenting role for single teen mothers.

### Comparing different family forms

An independent samples T-test was performed to compare parental efficacy and social support of single teen mothers across different family forms. Past research has proven that the two constructs influence each other [36–38]. Additionally, Raikes and Thompson [39] explained that when social support systems are weakened, parental efficacy diminishes as well. This shows that social support works as a mediator for parental efficacy. However, no studies attempted to look at parental efficacy and social support across different family forms, thus making comparisons between previous findings challenging. Nevertheless, the following results within the study showed that parental efficacy and social support was higher in extended family forms when compared to other family forms. However, when computing for guardian-skip generation, there was no relationship between parental efficacy and social support.

Furthermore, the results also indicated no correlation across subscales of social support. One study, did report findings on extended family, Johnson [40]. In particular, attachment to another parental figure other than the biological mother or father such as a grandmother or another relative saw these parental roles of extended family members as a surrogate parent and role model. This is very common within the Coloured communities, perhaps serving as a possible explanation to the majority of significant difference is found within this family form.

### Recommendations

Based from the results of this study, the following are recommended for future research:

1. Health care facilities, such as the MOU’s or counsellors, should consider a brief form of intervention in the form of creating a “PLAN” for expected teen mothers. This could help look at the confusion, challenges and changes that the teen mother would experience.
2. Develop and sustain NGO’s and agencies that can provide child care assistance for teen mothers who are working or going to school in relation to the child care grant. Perhaps create possibilities where help is offered in placing children of teen mothers in programs themselves.
3. Seek out possible options to help teen mothers and their families to realize that the pregnancy can be okay, as long as decisions are thought out and coping mechanisms are established and practiced. For example, foster support groups for teen mothers and their families.
4. Future researchers would benefit by including longitudinal and observational data investigating social support and parental efficacy could deeper the understanding of the association between the two constructs but more importantly their influence on teen mothers parenting development.

### Strength and limitations

We adapted questionnaires that have been validated and used by several researchers for this study. The results of the study should be understood with caution as the following limitations were documented. This study only focused on single teen mothers, residing in low socio-economic Coloured communities. Thus the findings would not be able to be generalized to a larger sample of single teen mothers, but only transferable to mothers who present similar characteristics and resides in comparable communities. In addition, the racial indication for the study was; Coloured 98.8%. The sample therefore could possibly suggest culture as a confounding variable.

## Conclusion

Single teen mothers whom reported high levels of parental efficacy, may have the confidence and beliefs within themselves that they are able to handle and successfully parent their child/ children. The positive relationships between social support and parental efficacy are important for planning and applying parenting programmes amongst single teen mothers and facilitating awareness regarding the importance of social support and family forms when considering parenting practices.

## Supplementary Information

**Additional file 1:** Mean (M) and standard deviation (SD) for single teen mother social support (N = 160), the social provision subscales (N = 160) and single teen mother parental efficacy (N = 160).

## Data Availability

More information on data from this study is available by contacting the corresponding author.

## Abbreviations

ANOVA: Analysis of variance
ATTACH: Attachment
GUIDE: Guidance
IBM: International Business Machines Corporation
M: Mean
MOUs: Midwife obstetric units
NGO: Non-profit organisation
NURTURE: Opportunity for nurturance
PE: Parental efficacy
PSOC: Parenting Sense of Competence Scale
REASSWORTH: Reassurance of worth
RELIABLE: Reliable alliance
SD: Standard deviation
SE: Standard error
SOCINTEGR: Social integration
SPS: Social Provisions Scale
SPSS: Statistical Package for the Social Sciences
SS: Social support
