# Supplementary material for: A comparison of the relationship between parental efficacy and social support systems of single teen mothers across different family forms in South African low socioeconomic communities
Source: BMC Womens Health. 2021 Apr 17;21:158. doi: 10.1186/s12905-021-01300-w (PMC8052653; doi:10.1186/s12905-021-01300-w)
Supplement: Supplementary file 1 — Additional file 1: Mean (M) and standard deviation (SD) for single teen mother social support (N = 160), the social provision subscales (N = 160) and single teen mother parental efficacy (N = 160). [file 12905_2021_1300_MOESM1_ESM.docx]

**Table S1: Mean (M) and Standard Deviation (SD) of items for Single Teen Mother Social Support (n=160)**

| Items  Social support | M | SD |
| --- | --- | --- |
| …I can depend on to help me if I really need it | 2.59 | 1.08 |
| …do not have close personal relationships with other people | 2.64 | 0.92 |
| …no one I can turn to for guidance in times of stress | 2.53 | 0.98 |
| …are people who depend on me for help | 2.54 | 0.83 |
| …are people who enjoy the same social activities I do | 2.63 | 0.88 |
| …people do not view me as competent | 2.61 | 0.76 |
| …feel responsible for the well-being of another person | 2.73 | 0.85 |
| …group of people who share my attitudes and beliefs | 2.61 | 0.81 |
| …other people respect my skills and abilities | 2.72 | 0.83 |
| …went wrong, no one would come to my assistance | 2.63 | 0.89 |
| …provide me with a sense of emotional security and well-being | 2.54 | 0.91 |
| …There is someone, I could talk to about important decisions in my life | 2.74 | 1.03 |
| …my competence and skills are recognized | 2.32 | 0.87 |
| …no one who shares my interests and concerns | 2.58 | 0.92 |
| …no one who really relies on me for their well-being | 2.57 | 0.95 |
| …could turn to for advice if I were having problems | 2.81 | 0.99 |
| …to have a strong emotional bond with at least one other person | 2.96 | 0.84 |
| …no one I can depend on for aid if I really need it | 2.58 | 0.97 |
| ...no one I feel comfortable talking about problems with | 2.65 | 1.00 |
| …people who admire my talents and abilities | 2.53 | 0.89 |
| …lack a feeling of intimacy with other people | 2.39 | 0.91 |
| …no one who likes to do the things I do | 2.47 | 0.91 |
| …people I can count on in an emergency | 2.74 | 1.01 |
| …no one needs me to care for them | 2.38 | 1.03 |

^Responses were on a Likert scale of 1= Strongly disagree, 2 = Somewhat disagree, 3 = Disagree, 4 = Agree., 5 = Somewhat agree, 6 = Strongly Agree. A high score indicates a greater degree of parental efficacy.^

**Table S2: Total Mean (*M*) and Standard Deviation (*SD*) scores for the Social Provision Subscales (N=160)**

| Variable | Min | Max | Mean | SD |
| --- | --- | --- | --- | --- |
| Attachment | 1.00 | 4.00 | 2.61 | 0.64 |
| Social Integration | 1.00 | 4.00 | 2.55 | 0.72 |
| Reassurance of Worth | 1.00 | 4.00 | 2.37 | 0.68 |
| Reliable Alliance | 1.00 | 4.00 | 2.53 | 0.81 |
| Guidance | 1.00 | 4.00 | 2.59 | 0.79 |
| Opportunity for Nurturance | 1.00 | 4.00 | 2.57 | 0.76 |

**Table S3: Mean (M) and Standard Deviation (SD) of items for Single Teen Mother Parental Efficacy (n=160)**

| Item  Parental Efficacy | M | SD |
| --- | --- | --- |
| …taking care of a child are easy to… | 2.86 | 1.69 |
| …could be rewarding. I am frustrated now while my child is at his/her present age | 4.47 | 1.47 |
| …wake up in the morning, feeling I have not accomplished a whole lot | 2.75 | 1.40 |
| …sometimes when I am supposed to be in control,  I feel more like the one being manipulated | 4.44 | 1.50 |
| My mother was better prepared to be a good mother than I am | 5.17 | 1.15 |
| …model for a new mother to follow in order to learn what she would need to know in order to be a good parent | 4.49 | 1.34 |
| …parent is manageable, and my problems are easily solved | 2.35 | 1.59 |
| …not knowing whether you doing a good job or a bad one | 4.94 | 1.29 |
| Sometimes I feel like I am not getting anything done | 4.65 | 1.46 |
| …personal expectations for expertise in caring for my child  in caring for my child | 3.68 | 1.19 |
| …find the answer to what is troubling my child, I am the one | 4.09 | 1.14 |
| …interests are in other areas, not being a parent | 4.07 | 1.43 |
| …I've been a mother, I feel thoroughly familiar with this role | 3.59 | 1.26 |
| …were only more interesting, I would be motivated to do a  better job as a parent | 4.43 | 1.43 |
| …all the skills necessary to be a good mother to my child | 3.64 | 1.29 |

^Responses were on a Likert scale of 1= Strongly disagree, 2 = Somewhat disagree, 3 = Disagree, 4 = Agree., 5 = Somewhat agree, 6 = Strongly Agree. A high score indicates a greater degree of parental efficacy.^
